# A Comparison of Copromicroscopic and Molecular Methods for the Diagnosis of Cat Aelurostrongylosis

**DOI:** 10.3390/ani12081024

**Published:** 2022-04-14

**Authors:** Simone Morelli, Donato Traversa, Anastasia Diakou, Mariasole Colombo, Ilaria Russi, Anton Mestek, Ramaswamy Chandrashekar, Melissa Beall, Barbara Paoletti, Raffaella Iorio, Athina Tsokana, Domitilla De Cristofaro, Alessandra Barlaam, Giulia Simonato, Angela Di Cesare

**Affiliations:** 1 Faculty of Veterinary Medicine, University of Teramo, 64100 Teramo, Italy; dtraversa@unite.it (D.T.); mcolombo@unite.it (M.C.); ilaria.russi.03@gmail.com (I.R.); bpaoletti@unite.it (B.P.); riorio@unite.it (R.I.); domitilla.decristofar@studenti.unite.it (D.D.C.); adicesare@unite.it (A.D.C.); 2 School of Veterinary Medicine, Faculty of Health Sciences, Aristotle University of Thessaloniki, 54124 Thessaloniki, Greece; diakou@vet.auth.gr; 3 IDEXX Laboratories Inc., Westbrook, ME 04092, USA; anton-mestekjr@idexx.com (A.M.); chandra-chandrashekar@idexx.com (R.C.); melissa-beall@idexx.com (M.B.); 4 Athina Vet Clinic, 84600 Ornos, Mykonos, Greece; athinatso2003@yahoo.gr; 5 Department of Agriculture, Food, Natural Resources and Engineering (DAFNE), University of Foggia, 71121 Foggia, Italy; alessandra.barlaam@unifg.it; 6 Department of Animal Medicine, Production and Health, University of Padua, 35020 Legnaro, Italy; giulia.simonato@unipd.it

**Keywords:** aelurostrongylosis, molecular, PCR, cat, pharyngeal swab, Baermann, diagnosis

## Abstract

**Simple Summary:**

Feline aelurostrongylosis is a worldwide distributed parasitic disease of domestic cats caused by the nematode *Aelurostrongylus abstrusus*. Cats may have a subclinical infection with this parasite or may show signs that overlap with those of many other respiratory conditions of cats, either parasitic or not. The diagnosis of feline aelurostrongylosis currently relies on fecal examinations, though this approach has some limits that can be overcome by innovative molecular techniques. Therefore, the present study has investigated the diagnostic reliability of a PCR assay performed on different samples collected from cats from Italy and Greece. The results of the study confirmed that PCR is highly sensitive and specific for the diagnosis of *A. abstrusus* infections in domestic cats.

**Abstract:**

The gold standard method for the diagnosis of cat aelurostrongylosis is the detection of *Aelurostrongylus abstrusus* first stage larvae with the Baermann’s examination. Nevertheless, molecular assays have shown higher diagnostic performances compared to copromicroscopy. This study evaluated the usefulness of an *A. abstrusus* species-specific PCR on different biological samples collected in clinical settings from 100 privately-owned cats in Italy (n. 60) and Greece (n. 40). A fecal sample was collected from each animal and a pharyngeal swab was also obtained for cats from Italy. All stool samples were subjected to flotation and Baermann’s test. The cats were categorized in three groups based on the results of copromicroscopy, i.e., Group A (n. 50 cats with *A. abstrusus* infection regardless of positivity for other helminths), Group B (n. 25 cats negative for *A. abstrusus* but positive for at least one of any other helminth), Group C (n. 25 cats negative for any helminth). DNA was extracted from individual aliquots of feces, flotation supernatant, Baermann’s sediment and the pharyngeal swab and then subjected to a PCR specific for *A. abstrusus*. At least one fecal aliquot or the pharyngeal swab scored positive by the *A. abstrusus*-specific PCR for 48/50 (96%) cats enrolled in Group A; in particular, 38/50 (76%), 35/50 (70%), 41/50 (82%) and 21/25 (84%) DNA extracts from feces, flotation supernatant, Baermann’s sediment and pharyngeal swabs were positive by PCR. These results confirm that molecular tools are highly sensitive and specific and indicate that pharyngeal swabs are the most suitable sample for molecular analysis in clinical settings.

## 1. Introduction

The cat lungworm *Aelurostrongylus abstrusus* (Nematoda, Metastrongyloidea) has become a priority in feline clinical medicine in many countries, due to its rediscovered pathogenic potential and a wide geographical distribution [1,2]. Cats acquire the parasite by ingesting infectious third stage larvae (L3) in mollusk intermediate or paratenic (e.g., small mammals, birds, reptiles or amphibians) hosts. After the infection and a migration which lasts less than 2 months, the parasite reaches adulthood inside bronchioles, alveolar ducts and alveoli [3,4,5]. After mating, females release eggs which hatch in the lung parenchyma and release first stage larvae (L1). These reach the pharynx via the mucociliary escalator, are swallowed and, via the feces, are excreted into the environment where they continue their development to L3 in terrestrial gastropods [4,6,7].

Infected cats display a range of clinical signs, from negligible/mild respiratory distress to a granulomatous pneumonia characterized by coughing, sneezing, oculo-nasal discharge, tachy-/dyspnea and life-threatening respiratory failure [2,8,9,10]. Nonetheless, the absence of respiratory signs does not rule out that infected cats have pulmonary damage and pathology [11,12,13].

Infected cats are often clinically undetected or misdiagnosed for several reasons, e.g., clinical signs may be inapparent, nonspecific, and/or overlap those of other feline diseases [2,14,15,16]. The current gold standard method for the diagnosis of *A. abstrusus* infection is the Baermann’s method, which is fairly sensitive, non-invasive and cost-effective. Nevertheless, fecal examinations have inherent limitations which can impair an accurate diagnosis [2,8,15], i.e., a minimum of 24 h is required to recover L1 in the Baermann’s apparatus, and the experience of the microscopist is crucial for larval detection and identification [2].

PCR-based tools can detect DNA molecules released from decaying cells of parasitic stages regardless of the presence of L1 in biological samples like feces or pharyngeal mucous [17,18,19]. Though molecular methods have shown increased diagnostic performance compared to conventional copromicroscopy, studies evaluating their sensitivity and specificity in large numbers of field cases are still lacking. The present work aimed at evaluating the usefulness of a PCR protocol specific for *A. abstrusus* when applied on different feline biological samples collected in clinical settings from varying geographical regions.

## 2. Materials and Methods

### 2.1. Study Cats and Samples

**Figure 1:**
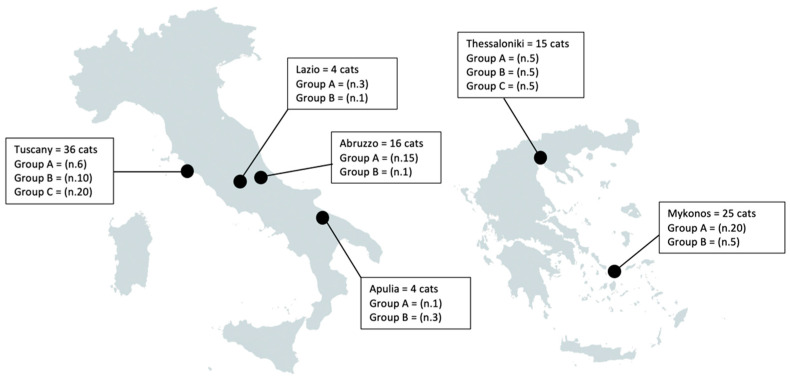
Study sites and number of cats enrolled in the study for each geographic area.

One hundred cats referred for routine procedures or clinical examinations to veterinary practices located in different Sites (Figure 1) of Italy (n. 60) and Greece (n. 40) were recruited in the study with the owner’s written consent. For cats enrolled in Italy, no ethical permission was required for routine clinical procedures herein performed, as per DM 4 March 2014 n. 26. For cats enrolled in Greece, the clinical procedures were approved by the Research Ethics Committee of the Aristotle University of Thessaloniki (Protocol No.: 215847/2021). An individual fecal sample was collected from each single animal and, from all cats from Italy, a pharyngeal swab was also obtained. Based on the results of copromicroscopy, cats were recruited until enrollment was complete for three convenience groups consisting of 50 cats infected by *A. abstrusus* at either flotation or Baermann’s test or both regardless of positivity for other helminthes (Group A), 25 cats negative for *A. abstrusus* at copromicroscopy but positive for at least one of any other helminth (Group B) and 25 cats negative for any helminth (Group C).

### 2.2. Conventional Examinations

All fecal samples were subjected to two copromicroscopic examinations, a flotation and the Baermann’s test, as follows. A classical flotation was performed on samples obtained from cats enrolled in Italy. Approximately 3 g of feces were mixed with 20 mL of sodium nitrate flotation solution (specific gravity 1.350), filtered through a sieve, put in a centrifuge tube (15 mL) and centrifuged at 600× *g* for 5 min. Thereafter, additional sodium nitrate flotation solution was added on the top of the tube, using a Pasteur pipette, until forming a meniscus and a coverslip was put on the top of the latter. After 5 min the coverslip was transferred to a glass slide and examined using a light microscope [20]. Fecal samples of cats recruited in Greece were examined using a different flotation procedure, i.e., the Faust method, as follows. Approximately 1 g of fecal material was diluted with tap water and passed through a sieve into a centrifuge tube. The tube was centrifuged at 200× *g* for 3 min, the supernatant fluid was discharged down to approximately 1 cm above the sediment and zinc sulphate solution 33.2% (*w/v*, specific gravity 1.18) was added to the sediment. After thorough dilution of the sediment, zinc sulphate solution was added over the top of the tube and a coverslip was placed on the top of the tube. After centrifugation at 150× *g* for 1 min, in a “swing-out” rotor centrifuge to retain the coverslip on the top of the tube, the coverslip was carefully removed, placed on a glass slide and examined using a light microscope [21].

The Baermann examination was performed following the same protocol for fecal samples of cats, both from Italy and Greece. Five to 10 g of feces were placed on a sieve and closed to form a pouch; the sample was placed in the Baermann apparatus, filled with room-temperature tap water. After 24 h, the sediment was collected in a centrifuge tube from the bottom of the apparatus and centrifuged at 600× *g* for 5 min. The supernatant was discarded, and the remaining sediment was collected with a Pasteur pipette and examined under a light microscope [22].

Individual aliquots of feces, flotation supernatant and Baermann’s sediment were collected and stored as previously described for further molecular examinations [17].

### 2.3. Molecular Analysis

DNA was extracted from fecal aliquots as in Section 2.2. and from pharyngeal swabs collected from cats from Italy. For fecal aliquots, DNA was extracted using the commercial kit “Exgene^TM^ Stool DNA minikit” (GeneAll^®^) using 200 µg for feces, 200 µL for flotation supernatant and 200 µL for Baermann sediment, respectively, by following the manufacturer’s instructions.

Prior to the extraction, pharyngeal swabs were immersed in an Eppendorf tube containing 1 mL of Phosphate Buffered Saline (PBS) and left over-night at room temperature. The swabs were removed from the tubes and placed in their original sheaths and centrifuged at 1000× *g* for 5 min. The sediment was collected and poured in the corresponding tube. All the tubes were centrifuged again at 19,000× *g* for 3 min, and the supernatant was discarded to obtain 200 μL of concentrated sample. From the latter, DNA was extracted using the commercial “DNEasy Tissue kit” (Qiagen GmbH, Hilden, Germany) [17].

All DNA extracts were subjected to a diagnostic nested PCR assay specific for a region internal to the ribosomal “Internal Transcribed Spacer 2” of *A. abstrusus* [17]. Briefly, the primers pairs NC1 (forward 5′ACGTCTGGTTCAGGGTTGTT3′) and NC2 (reverse 5′TTAGTTTCTTTTCCTCCGCT3′), and AabFor (forward 5′-GTAACAACGATATTGGTACTATG-3′) and AabRev (reverse 5′-GAACTCCTTCACGTGCTACTCG-3′) were used in the first and second step, respectively. The reaction mixture (50 µL) for the first step was prepared with 100 pmol of each primer (NC1-NC2), 5 µL of DNA, 25 mL of Red Taq Ready mix (Sigma Aldrich, St. Louis, MO, USA) and distilled water. The mixture for the second step was prepared using 200 pmol of each primer (AabFor and AabRev), 2 µL of a 1/20 dilution of NC1-NC2 of each amplicon, 25 mL of Red Taq Ready mix (Sigma Aldrich, St. Louis, MO, USA) and distilled water. For both steps, PCR was performed using an Applied Biosystems 2700 thermal cycler as follows: 94 °C for 7 min, 40 cycles and 94 °C for 45 s, 50 °C for 45 s, 72 °C for 45 s with a final extension phase at 72 °C for 10 min [17]. Larval *A. abstrusus* DNA and sterile water were included in every PCR run as positive and negative controls, respectively.

A dataset of 10 amplicons (~20% of the total) was selected for convenience to be directly sequenced.

### 2.4. Follow Up

Three cats from Italy were re-sampled 2 months after the first sampling for a further interpretation of doubtful results (see Section 3.3).

### 2.5. Sensitivity and Specificity

Cats were considered as true positives or negatives based on the results of the Baermann examination. The PCR results were compared with those obtained using the Baermann method for determining its sensitivity and specificity. The sensitivity and specificity of the PCR assay were determined as follows:

Sensitivity: number of cats positive at PCR for *A. abstrusus*/number of cats positive at copromicroscopy for *A. abstrusus*.

Specificity: number of cats negative at PCR for *A. abstrusus*/number of cats negative at copromicroscopy for *A. abstrusus*.

## 3. Results

### 3.1. Study Cats and Copromicroscopic Analysis

Cats infected by *A. abstrusus* in Group A scored either negative for other parasites (n. 30) or positive (n. 20), with varying percentages, for intestinal nematodes and/or tapeworms, and/or other lungworms. Moreover, cats in Group B were positive for different species of intestinal helminths and respiratory nematodes. Table 1 describes the number and provenance of cats enrolled in each single Group which were positive for different endoparasites, including *A. abstrusus*, at copromicroscopy.

### 3.2. Molecular Examination

At least one fecal aliquot or the pharyngeal swab scored positive with the *A. abstrusus*-specific PCR for 48/50 (96%) cats enrolled in Group A; in particular, 38/50 (76%), 35/50 (70%), 41/50 (82%) and 21/25 (84%) DNA samples from feces, flotation supernatant, Baermann’s sediment and pharyngeal swabs were PCR-positive. Non-specific amplicons were not produced for any of the samples obtained from cats which were infected by other endoparasites. All samples from Groups B and C were PCR-negative for amplicons of any size, except for 6 samples. These were 4 samples from Group B (i.e., 2 Baermann’s sediments, 1 Baermann’s sediment + flotation aliquot and 1 positive in all fecal aliquots) and two samples from Group C (i.e., pharyngeal swabs). Table 2 and Table 3 summarize the results of the molecular analysis of the samples collected from the 100 cats enrolled in the study. Ten sequences were obtained from the convenience dataset of amplicons (i.e., 6 and 4 from Group A and B, respectively) and all of them displayed a ~99–100% homology with the EU034168 *A. abstrusus* sequence.

### 3.3. Follow Up

Feces and mucus from 1 cat of Group B and 2 cats of Group C (see Section 3.2) were collected again after 2 months and re-examined by microscopy and PCR. The Baermann sediment of 1 cat of Group B (negative for *A. abstrusus* larvae at microscopy) scored positive for *A. abstrusus* at the first PCR examination. When re-sampled after two months, all biological samples of this cat tested negative at the PCR.

Two cats of Group C scored positive to *A. abstrusus* at the PCR on the pharyngeal swab. When the cats were sampled again after two months, the Baermann sediment of both was positive for *A. abstrusus* larvae (microscopic examination) and DNA (PCR). For both cats, the pharyngeal swab was again positive for *A. abstrusus* DNA.

### 3.4. Sensitivity and Specificity

The overall sensitivity of the PCR assay herein used was 96% (48/50), while the specificity was 94% (44/50).

## 4. Discussion

The present results confirm that the PCR is highly sensitive and specific for the diagnosis of feline aelurostrongylosis. Copromicroscopy has been herein considered the most reliable technique for the determination of the PCR sensitivity/specificity values, as it allows the direct visualization of parasites and is to date considered the “gold standard” for the diagnosis of patent feline aelurostrongylosis.

The first results obtained more than a decade ago proved that intraspecific nucleotide differences within the PCR target are not a constraint for the specific detection of *A. abstrusus* in a range of samples [17,18]. The data obtained herein from several *A. abstrusus* isolates collected in different geographic regions in two countries strongly confirm the reliability of this molecular method for diagnosing aelurostrongylosis in biological samples collected from cats living in different areas (Figure 1; Table 3). The PCR-positive cats (Table 3) with Baermann negative test results are most likely due to a factual absence of larvae in the examined stool. It may happen that L1 are not being shed even in cats with clinical signs and/or radiographic changes [2] due to the prepatent period of *A. abstrusus* or to the intermittent larval shedding in patent infections [23,24,25]. Moreover, *A. abstrusus* adults may survive for months after the infection in the lungs of cats which have ceased to excrete larvae, e.g., for immunological reasons [18,26]. In these cases, the molecular assay amplifies *A. abstrusus* water-soluble DNA molecules present in biological samples even in absence of larval shedding [17,18]. It should be considered that the sensitivity of the Baermann test is higher when performed on fecal samples collected over 3 days and using up to 10 g of feces [20]. Unfortunately, it was not possible to obtain three consecutive samples for the cats in this study, and this could have possibly influenced the copromicroscopic results of some cats in group B (see below).

Flotation results differ significantly between the two countries, as L1 of *A. abstrusus* (and of the closely related metastrongyloid *Troglostrongylus brevior*) were detected in a few samples from Italy while they were present in all samples collected from infected cats in Greece (Table 1). This shows that the copromicroscopic method (Faust flotation) used in Greece could be more sensitive than the conventional test used in Italy in detecting metastrongyloid L1. Most probably, the washing steps with water and the use of a more diluted flotation solution allows a successful detection of lungworm larvae with less chances of dehydration/rupture of the L1, as happens in the classic flotation procedure [2]. These data indicate that additional investigations are needed to further confirm if the Faust method used here could be considered a suitable alternative technique for the detection of lungworm L1.

Despite the differences in copromicroscopy, the results of the PCR performed on flotation aliquots from both countries had similar results. As previously shown [17], the Baermann sediment was the most suitable fecal substrate for the molecular detection of *A. abstrusus* (Table 2 and Table 3).

Though no pharyngeal swabs from cats enrolled in Greece were obtained, the results for swabs collected from cats of Group A in Italy confirm that these samples are highly suitable for the molecular diagnosis of feline aelurostrongylosis, with sensitivity values identical to those obtained for the Baermann sediment (Table 3).

The sequencing analysis, i.e., the positivity only for *A. abstrusus* of 19 fecal samples from cats co-infected with other parasites and the negativity of the vast majority of Group B and C cats, confirmed the high specificity of this nested PCR. Moreover, the method allowed the detection of occult aelurostrongylosis in 4 cats of Group B and 2 cats of Group C that had no copromicroscopic evidence of L1 in feces at the first examinations. The two cats of Group C that initially scored positive only to pharyngeal swab PCR were found positive for *A. abstrusus* L1 at the Baermann test (with molecular confirmation) when sampled a second time after two months. This indicates that pharyngeal swab molecular analysis can reveal aelurostrongylosis when L1 are not detected in the Baermann test, e.g., in prepatent infections, in cases with intermittent larval shedding, or when only a single fecal sample is examined. Unfortunately, additional samples were obtained only from 1 of the 4 PCR-positive cats of Group B. This cat had no evidence of *A. abstrusus* larvae and/or DNA when sampled for the second time. Most probably, this means that (i) at first sampling this cat was in a late/chronic stage of the infection with low parasitic burden, and/or (ii) a self-cure occurred when the cat was re-sampled. Accordingly, these considerations apply also for the other 3 cats of Group B whose feces scored positive for *A. abstrusus* DNA, which most likely had an occult aelurostrongylosis. If so, the specificity of the PCR used in the present study would have potentially been up to 100%.

The high sensitivity and specificity of this PCR have crucial implications under a clinical point of view. Indeed, other than the possibility to detect occult/pre-patent infections, this tool detects *A. abstrusus* DNA in presence of mixed infections, even when cats are co-infected with other lungworms such as the closely related *T. brevior* or the capillarid *Capillaria aerophila* (Table 2). The absence of cross-reactions with other lungworms is of key diagnostic and clinical relevance, as feline aelurostrongylosis, troglostrongylosis and pulmonary capillariosis may lead to completely different outcomes. As a mere example, severe clinical signs and fatal disease are usually more frequent in cats infected by *T. brevior* rather than in those infected by *A. abstrusus* or *C. aerophila* [2]. Most importantly, efficacious and on-label treatment options differ based on the lungworm species and should be adequately selected to guarantee both parasitological and clinical cure [2]. Therefore, a species-specific diagnosis is a mandatory first step for the most appropriate management of cats infected with lungworms. Moreover, the PCR can be indicated when a complete parasitological examination is not possible (e.g., low amounts of feces).

Although the molecular examination of the Baermann sediment and of the pharyngeal swabs have similar sensitivity values, the swab could be considered the best option in clinical settings for several reasons. This technique allows to overcome the difficulties in obtaining adequate fecal samples from cats. First, pharyngeal swabs can be more quickly obtained and prepared for DNA extraction, compared to the 12–24 h needed to obtain the migrating L1 from a Baermann examination [2]. Then, molecular analysis on DNA extracted from feces can be complicated by the presence of PCR inhibitors [17]. Moreover, DNA extracted from a pharyngeal swab would allow additional molecular tests for additional respiratory pathogens, which may cause clinical signs, similar to those induced by *A. abstrusus* (e.g., *T. brevior*, feline herpesvirus-1 (FHV-1), *Chlamydophila felis*, feline calicivirus (FCV) and *Mycoplasma felis*) [2,9,15,27].

In daily feline practice, the diagnosis of aelurostrongylosis in clinical settings is complicated by the lack of specificity of clinical signs, which may overlap those of parasitoses (e.g., troglostrongylosis, capillariosis, dirofilariosis and mycoses) or other non-parasitic diseases like feline asthma, chronic lower airway disease, tumours, bacterial or viral infections [2,9,15]. Moreover, abnormal radiographic findings in infected cats are nonspecific and may overlap those of many other diseases, thus representing another important limitation especially in those cats which are negative at copromicroscopy [2]. The gold standard method to detect *A. abstrusus* in infected cats, the Baermann’s migration method, has inherent drawbacks leading to false negative results and possible complications in the identification of larvae [2]. All cats showing even a single respiratory sign, especially if at-risk patients (outdoor access with potential ingestion of gastropods and/or small preys), should be subjected to appropriate copromicroscopic examinations, despite the limitation that negative results do not rule out the infection [8,25,28,29] (present results).

## 5. Conclusions

In conclusion, the results obtained in the present study confirm that molecular tools have the potential to overcome the limitations of the Baermann’s test for the diagnosis of the infection caused by *A. abstrusus*. A timely diagnosis is important also because it has been shown that damage to the lungs may already occur in early phases of infection, even before larvae are detected in the feces [30]. Thus, there is merit in investigating the diagnostic efficacy of rapid molecular techniques based on the here presented protocol, such as the Loop-Mediated Isothermal Amplification Assay (LAMP), i.e., a single tube and low-cost technique for the amplification of DNA, that do not require sophisticated equipment and that have already shown promise for the diagnosis of other nematode infections of veterinary relevance, e.g., *Ancylostoma caninum* [31] and *Toxocara* spp. [32].

## Figures and Tables

**Table 1:** Number, provenance and copromicroscopic findings of cats enrolled in the present study.

		Baermann		Flotation					
		*Aelurostrongylus abstrusus* n/tot (%)	*Troglostrongylus brevior* n/tot (%)	*Aelurostrongylus abstrusus* n/tot (%)	*Troglostrongylusbrevior* n/tot (%)	*Toxocara cati* n/tot (%)	Ancylostomatidae n/tot (%)	*Capillaria aerophila* n/tot (%)	*Toxascaris leonina* n/tot (%)
**Group A**	Italy	25/25 (100)	0/25 (0)	2/25 (8)	0/25 (0)	8/25 (32)	1/25 (4)	2/25 (8)	1/25 (4)
	Greece	25/25 (100)	7/25 (28)	25/25 (100)	7/25 (28)	1/25 (4)	2/25 (8)	1/25 (4)	0/25 (0)
	Total	50/50 (100)	7/50 (28)	27/50 (54)	7/50 (28)	9/50 (18)	3/50 (6)	3/50 (6)	1/50 (2)
**Group B**	Italy	0/15 (0)	0/15 (0)	0/15 (0)	0/15 (0)	8/15 (53.3)	6/15 (40)	1/15 (6.7)	0/15 (0)
	Greece	0/10 (0)	0/10 (0)	0/10 (0)	0/10 (0)	9/10 (90)	1/10 (10)	0/10 (0)	0/10 (0)
	Total	0/25 (0)	0/25 (0)	0/25 (0)	0/25 (0)	17/25 (68)	7/25 (28)	1/25 (4)	1/25 (4)
**Group C**	Italy	0/20 (0)	0/20 (0)	0/20 (0)	0/20 (0)	0/20 (0)	0/20 (0)	0/20 (0)	0/20 (0)
	Greece	0/5 (0)	0/5 (0)	0/5 (0)	0/5 (0)	0/5 (0)	0/5 (0)	0/5 (0)	0/5 (0)
	Total	0/25 (0)	0/25 (0)	0/25 (0)	0/25 (0)	0/25 (0)	0/25 (0)	0/25 (0)	0/25 (0)

n = number of positive animals; tot = number of examined animals.

**Table 2:** Positivity in a nested PCR species-specific for *Aelurostrongylus abstrusus* of different fecal aliquots and pharyngeal swab collected from cats of the present study. The different combinations of co-infections are detailed.

		Copromicroscopy n/tot (%)	PCR Feces n/tot (%)	PCR Flotation n/tot (%)	PCR Baermann n/tot (%)	PCR Pharyngeal Swab n/tot (%)
Italy						
Group A	*Aelurostrongylus abstrusus*	16/25 (64)	10/16 (62.5)	10/16 (62.5)	14/16 (87.5) *	14/16 (87.5)
	*Aelurostrongylus abstrusus* + *Toxocara cati*	7/25 (28)	4/7 (57.1)	6/7 (85.7)	6/7 (85.7) *	6/7 (85.7)
	*Aelurostrongylus abstrusus* + *Toxocara cati* + *Toxascaris leonina* + *Capillaria aerophila*	1/25 (4)	1/1 (100)	1/1 (100)	0/1 (0)	1/1 (100)
	*Aelurostrongylus abstrusus* + Ancylostomatidae + *Capillaria aerophila*	1/25 (4)	1/1 (100)	0/1 (0)	1/1 (100) *	0/1 (0)
Group B	*Toxocara cati*	8/15 (53.3)	0/8 (0)	1/8 (12.5)	2/8 (25) **	0/8 (0)
	Ancylostomatidae	6/15 (40)	0/6 (0)	0/6 (0)	0/6 (0)	0/6 (0)
	*Capillaria aerophila*	1/15 (6.7)	0/1 (0)	0/1 (0)	0/1 (0)	0/1 (0)
Group C	Negative at copromicroscopy	-	0/20 (0)	0/20 (0)	0/20 (0)	2/20 (10)
Greece						
Group A	*Aelurostrongylus abstrusus*	16/25 (64)	16/16 (100)	13/16 (81.3)	13/16 (81.3) *	NA
	*Aelurostrongylus abstrusus* + *Troglostrongylus brevior*	6/25 (24)	5/6 (83.3)	4/6 (66.7)	6/6 (100) *	NA
	*Aelurostrongylus abstrusus* + *Troglostrongylus brevior* + *Toxocara cati* + *Capillaria aerophila*	1/25 (4)	1/1 (100)	0/1 (0)	1/1 (100) *	NA
	*Aelurostrongylus abstrusus* + Ancylostomatidae	2/25 (8)	0/2 (0)	0/2 (0)	1/2 (50)	NA
Group B	*Toxocara cati*	9/10 (90)	1/9 (11.1)	1/9 (11.1)	2/9 (22.2) **	NA
	Ancylostomatidae	1/10 (10)	0/1 (0)	0/1 (0)	0/1 (0)	NA
Group C	Negative at copromicroscopy	-	0/5 (0)	0/5 (0)	0/5 (0)	NA
Total						
Group A	*Aelurostrongylus abstrusus*	32/50 (70)	26/32 (81.3)	23/32 (71.9)	27/32 (84.4)	14/16 (87.5)
	*Aelurostrongylus abstrusus* + *Troglostrongylus brevior*	6/50 (12)	5/6 (83.3)	4/6 (66.7)	6/6 (66.7)	NA
	*Aelurostrongylus abstrusus* + *Troglostrongylus brevior* + *Toxocara cati* + *Capillaria aerophila*	1/50 (2)	1/1 (100)	0/1 (0)	1/1 (100)	NA
	*Aelurostrongylus abstrusus* + *Toxocara cati*	7/50 (14)	4/7 (57.1)	6/7 (85.7)	6/7 (85.7)	6/7 (85.7)
	*Aelurostrongylus abstrusus* + *Toxocara cati* + *Toxascaris leonina* + *Capillaria aerophila*	1/50 (2)	1/1 (100)	1/1 (100)	0/1 (0)	1/1 (100)
	*Aelurostrongylus abstrusus* + Ancylostomatidae	2/50 (4)	0/2 (0)	0/2 (0)	1/2 (50)	NA
	*Aelurostrongylus abstrusus* + Ancylostomatidae + *Capillaria aerophila*	1/50 (2)	1/1 (100)	0/1 (0)	1/1 (100)	0/1 (0)
Group B	*Toxocara cati*	17/25 (68)	1/17 (5.9)	2/17 (11.8)	4/17 (23.5)	0/17 (0)
	Ancylostomatidae	7/25 (28)	0/7 (0)	0/7 (0)	0/7 (0)	0/7 (0)
	*Capillaria aerophila*	1/25 (4)	0/1 (0)	0/1 (0)	0/1 (0)	0/1 (0)
Group C	Negative at copromicroscopy	NA	0/25 (0)	0/25 (0)	0/25 (0)	2/20 (10)

* One amplicon subjected to sequencing; ** amplicons subjected to sequencing; n = number of positive animals; tot = number of examined animals; NA = Not Applicable, pharyngeal swabs were collected only for cats enrolled in Italy.

**Table 3:** Overall positivity in a nested PCR species-specific for *Aelurostrongylus abstrusus* of different fecal aliquots and pharyngeal swab collected from cats of the present study.

	PCR Feces n/tot (%; 95% CI)	PCR Flotation n/tot (%; 95% CI)	PCR Baermann n/tot (%; 95% CI)	PCR Pharyngeal Swab n/tot (%; 95% CI)	Total PCR n/tot (%; 95% CI)
Italy					
Group A	16/25 (64; 42.5–82)	15/25 (60; 38.7–78.9)	21/25 (84; 63.9–95.5)	21/25 (84; 63.9–95.5)	24/25 (96; 79.7–99.9)
Group B	0/15 (0)	1/15 (6.7; 0.2–32)	2/15 (13.3; 1.7–40.5)	0/15 (0)	2/15 (13.3; 1.7–40.5)
Group C	0/20 (0)	0/20 (0)	0/20 (0)	2/20 (10; 1.2–31.7)	2/20 (10; 1.2–31.7)
Greece					
Group A	22/25 (88; 68.8–97.5)	18/25 (72; 50.6–87.9)	20/25 (80; 59.3–93.2)	NA	24/25 (96; 79.7–99.9)
Group B	1/10 (10; 0.3–44.5)	1/10 (10; 0.3–44.5)	2/10 (20;2.5–55.6)	NA	2/10 (20;2.5–55.6)
Group C	0/5 (0)	0/5 (0)	0/5 (0)	NA	0/5 (0)
Total					
Group A	38/50 (76; 61.8–86.94)	35/50 (70; 55.4–82.1)	41/50 (82; 68.6–91.4)	21/25 (84; 63.9–95.5)	48/50 (96; 86.3–99.5)
Group B	1/25 (4; 0.1–20.4)	2/25 (8; 1–26)	4/25 (16; 4.5–36.1)	0/15 (0)	4/25 (16; 4.5–36.1)
Group C	0/25 (0)	0/25 (0)	0/25 (0)	2/20 (10; 1.2–31.7)	2/25 (8; 1–26)

n = number of positive animals; tot = number of examined animals; 95% CI = 95% Confidence Interval; NA = Not Applicable, pharyngeal swabs were collected only for cats enrolled in Italy.

## Data Availability

All the data generated are described in the manuscript.
